# Urban rats have less variable, higher protein diets

**DOI:** 10.1098/rspb.2018.1441

**Published:** 2018-10-17

**Authors:** E. Guiry, M. Buckley

**Affiliations:** 1 Department of Anthropology, Trent University, 1600 W Bank Drive, Peterborough, Ontario, Canada K9 J 0G2; 2 Department of Anthropology, University of British Columbia, 6303 NW Marine Drive, Vancouver, British Columbia, Canada V6T 1Z1; 3 School of Earth and Environmental Sciences, Manchester Institute of Biotechnology, University of Manchester, 131 Princess Street, Manchester M1 7DN, UK

**Keywords:** urban ecology, archaeology, commensalism, *Rattus norvegicus*, stable isotopes, historical ecology

## Abstract

Over the past 1000 years, rats (*Rattus* spp.) have become one of the most successful and prolific pests in human society. Despite their cosmopolitan distribution across six continents and ubiquity throughout the world's cities, rat urban ecology remains poorly understood. We investigate the role of human foods in brown rat (*Rattus norvegicus*) diets in urban and rural areas over a 100 year period (*ca* AD 1790–1890) in Toronto, Canada using stable carbon (*δ*^13^C) and nitrogen (*δ*^15^N) isotope analyses of archaeological remains. We found that rat diets from urban sites were of higher quality and were more homogeneous and stable over time. By contrast, in rural areas, they show a wide range of dietary niche specializations that directly overlap, and probably competed, with native omnivorous and herbivorous species. These results demonstrate a link between rodent diets and human population density, providing, to our knowledge, the first long-term dietary perspective on the relative value of different types of human settlements as rodent habitat. This study highlights the potential of using the historical and archaeological record to provide a retrospective on the urban ecology of commensal and synanthropic animals that could be useful for improving animal management and conservation strategies in urban areas.

## 1. Introduction

Rats (*Rattus* spp.) have played an important role in many dimensions of human life. Considerable attention has been paid to how the global dissemination of rats, especially black (*Rattus rattus*), brown (*Rattus norvegicus*), and Pacific (*Rattus exulans*) rats, have been implicated in broad scale environmental destruction [1], the spread of deadly zoonotic diseases that pose significant global health risks [2], and the billions of dollars spent annually on pest control [3,4]. Beyond a general understanding of how rats have followed humans owing to the valuable habitat (i.e. food and shelter) that human-structured ecosystems provide, relatively little is known about how and why rats have been so successful at exploiting their relationship with humans at different temporal and spatial scales [5]. In this context, the history of rat dissemination remains sketchy and basic questions persist unanswered, such as, when, where, and which rat populations were involved in early migrations and specifically what aspects of human settlement were most facilitative to their spread [6,7]. Moreover, despite centuries of cohabitation with rats in urban areas, modern ecological studies have yet to establish a unified understanding of urban rat ecology [6].

Very little information is available on the ecology of past rat populations because much of the early spread of rats in human settlements occurred prior to the development of scientific observation. However, because rats were often one of the first invasive mammalian species to be introduced by humans to many regions of the globe, a better understanding of their early ecology may hold significant potential for addressing larger questions about their impact on novel ecosystems. For instance, what impacts did rat introductions, as opposed to direct human exploitation or habitat destruction, have on the extirpation or extinction of local wild taxa? What behavioural modifications have indigenous taxa undergone to adapt to new competition with incoming rat species? Moreover, rats, as initial colonizers of new ecosystems, could have the potential to serve as a comparator and/or a model system for a range of other commensal species such as house mice or fruit flies. For this reason, a better understanding of the mechanisms that facilitated the successful co-global colonization of rat species could be used as an analogy for other, less accessible (archaeologically or otherwise) commensal species by providing improved models for assessing which dimensions the human-commensal relationship are most salient for the spread of anthrodependent species.

In the context of historical ecology and conservation biology, researchers in adjacent fields are increasingly relying on chemical and genetic analyses of historical museum-archived specimens to address questions of species behaviour and long-term environmental variation, especially in the context of understanding how biological communities have adapted to human-altered environments [8,9]. This historical approach could provide a valuable retrospective on how rats have engaged with and adapted to different kinds of anthropogenic systems. Unfortunately, urban rat populations have rarely been the focus of historical or long-term ecological research and historical specimens are therefore rarely present in large quantities in natural history collections. Fortunately, archaeological rat remains could represent a valuable archive of specimens dating to the earliest timeframes of rat–human cohabitation and can provide an invaluable resource for biomolecular research (e.g. isotopic, aDNA). Rat remains are, for instance, commonly found in historical archaeological deposits dating back to some of the earliest European occupations in the New World [10], and on shipwrecks [11] associated with the first arrivals of European settlers to the Americas.

Because food is at the heart of rat–human commensalism, isotopic analyses of rat remains has outstanding potential to illuminate a variety of aspects of early rat ecology and human behaviour. Isotopic analyses of archaeological rat bones can be used to reconstruct patterns in what foods were available to rats in human settlements through time [12] and across space [13]. In addition to providing a baseline for the kinds of food that rats scavenged [14], these data also provide details on how broader cultural (socio-economic shifts related to food production) [12,15] and environmental (impacts on landscapes and native taxa) [13] processes unfold. Moreover, isotopic analyses of archaeological rat remains may be used to reveal patterns in where and how rats have been most successful at exploiting human settlements. In turn, this information could provide a long-term retrospective in which the dynamics of rat infestations in the modern world can be contextualized and better understood.

We approach the question of how early rat–human relationships in urban environments unfolded from the perspective of rat diets. Using collagen peptide mass fingerprinting, also known as zooarchaeology by mass spectrometry (ZooMS) [16], combined with isotopic analyses of bone collagen from 86 archaeological brown rats (*R. norvegicus*; hereafter referred to as ‘rats’), we assess differences in diet between rat populations from urban and rural habitats as a proxy for relative dependency on human food systems in nineteenth-century Upper Canada. Our working hypothesis is that rat diets will incorporate more high quality foods (including animal protein) in denser human settlements, whereas rats foraging in less human-structured habitats in rural and peripheral settings will have less frequent access to higher quality foods. By comparing stable carbon (*δ*^13^C) and nitrogen (*δ*^15^N) isotopic compositions of ZooMS-confirmed rat bone collagen from sites associated with different kinds and intensities of human activity, this research aims to investigate the relationship between rat behaviour and human population density in two key areas: (i) the ways in which rat diets differ with proximity to human settlement density; and (ii) whether rats living among denser urban populations enjoy higher quality diets (i.e. diets incorporating more animal fats and protein) more consistently than their rural counterparts.

## 2. Context, materials, methods

### (a) Methodological context

The isotopic composition of collagen from archaeological rat bones can provide information about the kinds of foods these animals consumed (for a review of stable isotopes in archaeology see [17]). Because bone collagen remodels slowly over the life of an individual, the isotopic composition of a rat bone will reflect a long-term average of dietary intake over the lifespan of the individual [18]. For nineteenth-century Upper Canada rural and urban settlements, we expect that rat *δ*^15^N values should primarily reflect the trophic level at which an individual fed [19], with lower values suggesting a more herbivorous diet and higher values suggesting increased omnivory (i.e. consumption of more meat and other animal products). Because *R. norvegicus* is known to be more omnivorous and to preferentially select energy rich foods (particularly meat) when available [20,21], their *δ*^15^N values can serve as a proxy for diet quality. Rat *δ*^13^C values provide an indicator for the extent to which they had access to foods derived from C_3_ or C_4_ plants [22]. In the context of nineteenth-century Upper Canada, most foods were derived from C_3_ plants, however, it is possible that maize (a major C_4_ crop) or meat from maize-fed animals could have been available through trade [23].

### (b) Sample description

**Figure 1.**
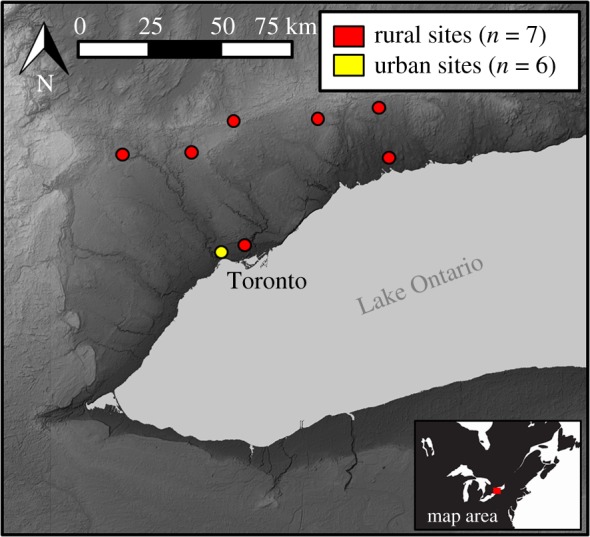
Map showing locations of nineteenth-century (AD 1790–1890) archaeological sites from York (now Toronto), Upper Canada (now Ontario, Canada). (Online version in colour.)

All samples included in this study come from archaeological deposits at 13 sites (figure 1) in the immediate vicinity of the present day city of Toronto, Canada (i.e. within 75 km) and date to the same historical period (AD 1790–1890) of intensification of European settlement in the Lower Great Lake region (see the electronic supplementary material, table S1). To avoid duplicating results from the same individual, samples were selected based on minimum number of individual counts per archaeological context. The urban assemblage of rat bone samples (*n* = 42) comes from five houses and a hospital in the city of York (now Toronto), in Upper Canada (now southern Ontario, Canada) whereas the rural assemblage of rat bone samples (*n* = 44) comes from seven sites located up to 75 km from York (figure 1). A range of environmental and cultural processes can contribute to variation in *δ*^15^N and *δ*^13^C values at the base of the food web (e.g. [24,25]), which can result in spatio-temporal variability in the isotopic composition of consumer tissues [9,26]. For this reason it is critical that rat isotopic compositions are interpreted relative to an isotopic baseline from other species that are from the same time period and local to the study area [27]. Therefore, we also analysed coeval late eighteenth- to late nineteenth-century specimens from local native omnivores (racoons, *Procyon lotor*; *n* = 6) and herbivores (groundhogs, *Marmota monax*; *n* = 8) from the same region sites to establish a baseline for local wild fauna. To provide a baseline for locally husbanded domestic animals we used previously published data from archaeological livestock (*n* = 286) from the same region (including some of the same sites) and time period [23].

### (c) Sample preparation

Bones were cleaned of surface contamination and cut into small pieces (approx. 3 mm^3^). Samples were demineralized over several days in 0.5 M hydrochloric acid (HCl). Demineralized samples were then neutralized in type I water. To remove base-soluble contaminants resulting from diagenetic processes in the archaeological burial environment, samples were subjected to a series of 0.1 M sodium hydroxide (NaOH) treatments in an ultrasonic bath (NaOH solution refreshed every 15 min) until solution remained clear [28]. Collagen was then solubilized in a pH3 solution (pH adjusted with HCl) for 48 h at 65°C. Samples were then centrifuged and the solubilized collagen fraction was transferred to a new tube, frozen, and lyophilized in a freeze dryer.

### (d) Isotopic analyses

Isotopic compositions were measured on 0.5 mg collagen subsamples in tin capsules using an Elementar Vario MICRO cube elemental analyser coupled via continuous flow to an Isoprime isotope ratio mass spectrometer in the Archaeology Chemistry Laboratory at the University of British Columbia, Canada, with duplicate analyses being performed on 13% of samples. Calibration and analytical uncertainty is described in the electronic supplementary material. Measured *δ*^13^C and *δ*^15^N values were calibrated relative to Vienna Pee Dee Belemnite (VPDB) and ambient inhalable reservoir (AIR) respectively using a two point calibration curve anchored to United States Geological Survey (USGS) 40, USGS 41 and USGS 41a [29] (electronic supplementary material, table S2). The accuracy of isotopic measurements was assessed using internal check standards (electronic supplementary material, table S3). For check standards the average absolute difference between observed and known *δ*-values (i.e. reproducibility or accuracy) was 0.03‰ for *δ*^13^C and 0.02‰ for *δ*^15^N. For all standards (1*σ* of check and calibration), measurement precision was ±0.05‰ and ±0.10‰ for *δ*^13^C and *δ*^15^N respectively. The average difference between duplicate sample pairs was 0.01‰ and 0.05‰ for *δ*^13^C and *δ*^15^N, respectively. Analytical uncertainty was ±0.10‰ and ±0.12‰ for *δ*^13^C and for *δ*^15^N, respectively [30] (see the electronic supplementary material). Stable isotope compositions of bone collagen are considered acceptable when accompanied by the following data on collagen integrity criteria [31]: (i) per cent C and N values above 18% and 6%, respectively; and, (ii) atomic C : N values falling between 2.9 and 3.6. In order to ensure appropriate statistical methods for comparing data were applied, we assessed the distribution of our data using Shapiro–Wilk's, Mann–Whitney–Wilcoxon's and, Levene's tests. Statistical analyses for determining difference in means and variance between rat populations were conducted using R (version 3.4.3; Shapiro–Wilk test and Mann–Whitney–Wilcoxon test) as well as the R package car (Levene's test) [32]. For random comparisons between groups the dplyr package was used. To compare niche size between rat populations we calculated their convex hull areas (TA), an area based metric used to quantify niche widths, using the R package, Stable Isotope Bayesian Ellipses in R (SIBER) [33]. A convex hull is the smallest convex polygon in bivariate isotopic space that contains all data within a group, and provides a basis for quantifying and comparing the relative extent of dietary diversity between two or more populations with similar sample sizes [34].

### (e) Zooarchaeology by mass spectrometry analyses

Rat dietary behaviour can be influenced by interspecific competition between *Rattus* species [35] and, for this reason, understanding the taxonomic composition of our rat sample is important for interpretations of urban and rural rat ecology [12]. While it is thought that the early dominance of the brown rat (*R. norvegicus*) prevented the black rat (*R. rattus*) from becoming established in the region [36], there remained a possibility that our rat sample could potentially include multiple species of *Rattus*. Therefore, we used ZooMS to provide species-level taxonomic determination [37,38] for the majority of samples. In brief, several milligrams of collagen per sample, resuspended in 50 mM ammonium bicarbonate, was digested with 0.4 µg sequencing grade trypsin for 18 h at 37°C before being acidified to 0.1% trifluoroacetic acid (TFA). Peptide digests were then applied to an equilibrated C18 solid phase extraction cartridge, washed twice with 0.1% TFA, and fractionated into 10% and 50% (acetonitrile (ACN) in 0.1% TFA) fractions following [16]. These fractions were then evaporated and resuspended with 10 µl 0.1% TFA and 1 µl co-crystalized with an equal volume of 10 mg ml^−1^ alpha-cyano hydroxycinnamic acid in 50% ACN/0.1% TFA and allowed to air dry. Collagen peptide mass fingerprint spectra were then acquired using a Bruker Ultraflex II Matrix Assisted Laser Desorption Ionization Time of Flight (MALDI-ToF) mass spectrometer with up to 2000 laser acquisitions over the *m/z* range 700–3700. Spectra were then compared with those of rats published previously [38] as well as those of other rodents (e.g. [37,39]). Of particular significance to this work was distinguishing between the two expected *Rattus* species through observation of biomarkers at either *m/z* 2957.4 or *m/z* 2987.4, when supported with other *Rattus* markers such as those at *m/z* 1451.7 and *m/z* 2143.1 (electronic supplementary material, figure S1).

## 3. Results

**Figure 2.**
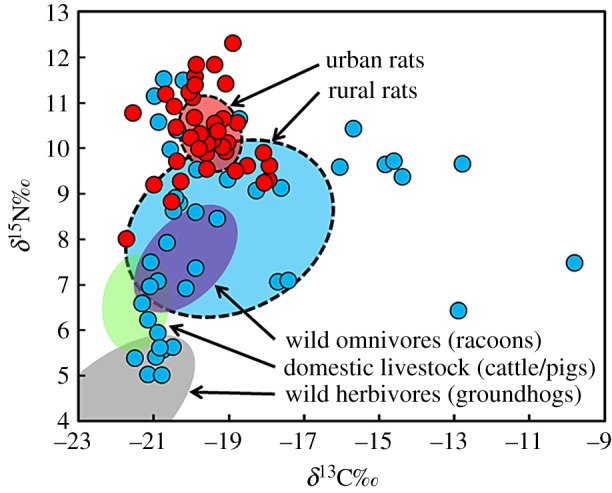
Plot comparing all data included in this study. Urban (*n* = 42) and rural (*n* = 44) rat *δ*^13^C and *δ*^15^N values are shown as red and blue circles respectively. Standard ellipses for urban (red ellipse) and rural (blue ellipse) rats and other animal groups are also shown for comparison: purple ellipse is wild omnivores (six racoons), green ellipse is livestock (256 cattle and pigs) [23] and grey ellipse is wild herbivores (eight groundhogs). A plot comparing all *δ*^13^C and *δ*^15^N values for individual animals from all taxa is provided in the electronic supplementary material, figure S2. (Online version in colour.)

The majority (85%) of rat samples provided collagen peptide mass fingerprint spectra of sufficient quality for species-level determinations (electronic supplementary material, table S1), all but one of which were *R. norvegicus*, and this provides strong support for the idea that *R. rattus* was not historically present in the region. The non-*Rattus* specimen was removed from analyses. The isotopic values of rat bone collagen (figure 2; electronic supplementary material, table S2) were interpreted in the context of a large isotopic baseline for contemporaneous wild omnivores (racoons, *n* = 6; *δ*^13^C = −20.2 ± 1.2‰, *δ*^15^N = +7.6 ± 1.1‰) and herbivores (groundhogs, *n* = 8; *δ*^13^C = −22.0 ± 2.1‰, *δ*^15^N = +4.4 ± 1.6‰) as well as livestock from the same region; these included cattle (*n* = 152) and pigs (*n* = 114), which serve as a baseline for locally produced meat products (*n* = 256; *δ*^13^C = −21.5 ± 0.9‰, *δ*^15^N = +6.6 ± 1.1‰) [23]. Analyses show that rats from urban archaeological sites (*n* = 41) in the historic settlement of York in Upper Canada consistently had the highest *δ*^15^N (+10.3 ± 0.9‰; range = 4.6‰) and lowest *δ*^13^C (−19.6 ± 0.9‰; range = 3.8‰) values with relatively little variation. These data show that rats living in urban habitats had regular access to higher quality (animal) protein sources. Meanwhile rats from seven rural sites (*n* = 45) show highly variable and widely ranging *δ*^15^N (+8.2 ± 1.9‰; range = 6.5‰) and *δ*^13^C (−19.0 ± 2.7‰; range = 9.8‰) values, consistent with a habitat and diet where broader foraging strategies were needed and less reliable food subsidies from human food systems were available. A Shapiro–Wilk test was used to determine that for both urban and rural rat populations, *δ*^13^C values were normally distributed (for urban rats, *n*= 41, *W* = 0.76647, *p* ≤ 0.000; for rural rats, *n* = 44, *W* = 0.975, *p* = 0.492) whereas *δ*^15^N values were not (for urban rats, *n*= 41, *W* = 0.950, *p* = 0.053; for rural rats, *n* = 44, *W* = 0.986, *p* = 0.878). Levene's and Bartlett's tests were therefore used to determine homogeneity of variance for *δ*^13^C and *δ*^15^N values respectively, and showed that group variances were unequal (Levene's test, *F*_84_ = 8.8192, *p*= 0.003; Bartlett K-squared = 23.347, d.f. = 1, *p ≤* 0.000). Because variances were not equal, a Mann–Whitney–Wilcoxon test was used to compare the difference in means between rat groups. While urban and rural rat populations have significantly different *δ*^15^N values (*W* = 348, *p ≤* 0.000), their *δ*^13^C values do not differ significantly *(W* = 774, *p* = 0.198). Convex hull areas calculated in SIBER for groups of rats show large differences in isotopic niche width, confirming that the rural rat (TA = 47.6) population had much more (over than four times more) dietary niche diversity than their urban (TA = 10.1) counterparts. A similarly large range of intra-site rat isotopic variation can be found at multiple rural sites, suggesting that differing patterns in isotopic variation between rats at urban and rural sites are not related to spatial variables associated with differing local geographies. We also assessed this possibility statistically by bootstrapping 20 samples from urban and rural groups of rats 10 times and comparing their *δ*^15^N values with Mann–Whitney–Wilcoxon's tests. Results confirm (for 9 of 10 tests, *p ≤* 0.05; see the electronic supplementary material, table S5) that the urban and rural rat groups are significantly different regardless of sites included.

All rats analysed in this study come from the same general time period (i.e. AD 1790–1890); however, narrower date ranges, based on archaeological context, are available for specimens from some sites. Unfortunately, these date ranges are relatively coarse (spanning between three and nine decades) and therefore only permit assignment of samples to broad and often overlapping temporal categories (i.e. earlier, middle or later nineteenth century). Although the date ranges offered by archaeological context information do not provide the temporal resolution that would be necessary for statistical comparison between time periods, they can, nonetheless, allow for some general observations. While the majority of rats come from archaeological contexts with date ranges spanning the entire timeframe of this study, both urban and rural datasets include a smaller subset of rat samples from archaeological contexts dating from the early-mid and mid-late nineteenth century. For urban rats, these earlier and later nineteenth century subgroups show a similar degree of isotopic variability suggesting that the relative amount of dietary variation for urban rats was consistent through time. Unfortunately, for rural rats, there are too few specimens from narrowly dated archaeological contexts to meaningfully compare time periods. However, given that higher intra-site variation occurs in rat isotope values at multiple sites, and that most rural sites span the entire nineteenth century, we expect that dietary heterogeneity was relatively constant for rural rat populations through time.

## 4. Discussion and conclusion

This study is, to our knowledge, the first to use isotopic analyses of archaeological fauna to compare urban and rural rodent ecology though time. The isotopic composition of nineteenth-century rat remains from Upper Canada indicates that brown rat populations living within denser human settlement areas probably fared better, from a dietary perspective (i.e. had diets richer in animal-based foods), than those living further away. In the context of modern ecological research on rats, suggesting that diet quality and association with anthropogenic environments can result in greater fecundity and fitness [40,41], these findings serve not only to highlight a close relationship between rat ecology and urbanism, but also can provide a framework for testing archaeological questions about commensality and urbanization.

The long-term relationship (generally spanning AD 1790–1890) observed here between settlement type and rat diet offers a new perspective on the past ecology of urban rodents and could provide a potentially valuable indicator for urbanization in archaeological contexts. Because rat populations inhabiting denser urban environments have access to a larger range of food sources (e.g. [6,42,43]), more individuals should have opportunities to obtain preferred higher quality food items that, particularly for *R. norvegicus*, can emphasize animal protein/fat sources [20,21]. Our analyses show that this scenario can result in rat diets that, at the population level, are more homogeneous isotopically and consistently include greater proportions of higher trophic level food inputs. By contrast, in less dense settlements human-derived food may be scarcer, leading to rat diets that include lower trophic level foods with greater inter-individual dietary specialization. The association between settlement density and diet quality/variation could therefore provide a marker for degree urbanization (i.e. higher diet quality and lower dietary variation equals greater urbanization). Such a proxy measure could be useful as an independent perspective for tracking settlement density at archaeological sites where rat remains are available from a range of time periods. Further investigation of this possibility, through additional comparisons of rat isotopic compositions at other urban and rural locations, would help to establish the extent to which this dietary relationship is characteristic of rat diets across sites with different human settlement densities.

It is also noteworthy that for rural environments our data show that in some cases, at the individual level, rats still specialized in taking advantage of human food systems. In particular, a small number of rural rat *δ*^13^C values suggest their activity in the pilfering of maize, a C_4_ plant that was a significant crop for European settlers and migrants in many parts of eastern North America during the eighteenth and nineteen centuries, but not in Upper Canada [44]. This is interesting because it demonstrates a pattern that is not shown by the isotopic composition of livestock from the same sites [23]. In the context of nineteenth-century Upper Canada, analyses of herbivores that are probably locally sourced confirm that C_4_ plants such as maize were not commonly used as a fodder source. Elevated *δ*^13^C values from select rats at multiple rural sites therefore suggest that some C_4_ materials, probably maize based on historical context, could have been present at rural farm sites. Because *δ*^15^N baseline values can vary over space and time [9,24], the wide variability in corresponding *δ*^15^N values for these C_4_-feeding rats is difficult to interpret and rat consumption of imported meat products derived from maize-fed animals cannot be ruled out. In the context of historical and archaeological data showing that animal products consumed at the same sites derived mainly from C_3_-fed animals and that C_4_ plants were probably not cultivated for animal feed, the most parsimonious interpretation of these C_4_ rat data is that rats had access to stores of imported maize that was kept on hand for human consumption or perhaps for tasks such as feeding domestic fowl.^1^ It is also possible that C_4_-feeding rats could represent intrusive individuals from more recent twentieth-century rat populations, although this explanation is less parsimonious given the fact that these C_4_-feeding individuals are observed at multiple sites and in a range of well dated archaeological contexts.

Archaeological data from urban rats can also provide insight into the question of why rats have been so successful at exploiting human environments. Because archaeological rat specimens come from multiple sites in different areas of the city and represent animals that lived at different times throughout the nineteenth century, the relatively low degree of isotopic variation suggests that the dietary ecology of urban rats was stable for a long period of time. This suggests that not only has the urban habitat of York provided rats with higher quality foods, but that it had done so consistently over a century. In considering the cost to commensals of inhabiting anthropogenic environments, Hulme-Beanam and colleagues have suggested that, while urban environments today can provide a more steady supply of food to commensal animals, past urban commensal populations would probably have experienced greater variation in food supply [7] and that this unpredictability would have had evolutionary consequences for species dependent on human food systems. Our results suggest that, in at least some areas of the world, food supply in urban settlements was sufficiently abundant and stable to allow for dietary consistency since at least the nineteenth or even the late eighteenth century. From a pest management perspective, the apparent dietary consistency of urban rats suggests that one way of reducing the quality of urban habitat for rats could be disruption, at least periodically, of higher trophic level food supplies. While it is possible that our urban sample is somehow systematically biased to include more rat specimens from particular time periods in which better quality foods were available, our sampling strategy was random and aimed to incorporate rat remains from as many spatiotemporally varied contexts as possible. Therefore the complete lack of individuals with lower trophic level diets in the urban assemblages suggests that this pattern is not greatly influenced by archaeological preservation biases. In this context, we interpret higher quality and more homogeneous rat diets as evidence for a close adaptation between rat ecology and urban environments, in turn, providing support for the idea that the success of rats, and perhaps other urban commensals, in human settlements stems at least partly from the long-term reliability of human food systems.

The retrospect offered by archaeological rat remains may also be able to shed light on the dietary dimensions and evolutionary nature of rat commensality. Banks & Smith [45] have recently argued that some species of rats, *R. rattus* in particular, are native to urban areas and are in fact only alien once they colonize peri-urban and rural/peripheral environments. This assertion is based not only on the firm association of certain rat species with human settlements, but also on the knowledge that these species are no longer known to exist in a wild state [46]. This idea is supported by our findings, suggesting that rats in urban locations had better and more consistent access to higher quality foods than their rural counterparts, which underwent a range of dietary niche specializations.

From an ecological perspective, the high degree of isotopic variation observed in rats from rural sites can help to better contextualize ecological consequences of rat introductions. Archaeological rats from rural environments adopted a diversity of specialized feeding behaviours that would have put them in competition for niches contemporaneously occupied by native fauna. In this study, for instance, we observe that whereas rats from urban contexts show no overlap with wild taxa (figure 2), rural rats show considerable overlap with both wild herbivores and omnivores, suggesting that racoons, groundhogs, and other rodents may have experienced increased competition for food sources from rat introductions. Given the early dates from some of these specimens (i.e. as early as the later eighteenth century), this archaeological evidence adds considerable temporal depth to the observation that commensal rodent introductions can directly influence native fauna through competition for habitat and food resources.

This study has sought to highlight the potential value of archaeological rat remains as a resource for developing long-term perspectives on urban rat ecology. In the past, researchers have argued that current trends in human population increase [47], urbanization [48], and climate change [49] will serve to significantly expand availability of favourable human-structured habitat for rats. These studies point out that effective mitigation of the risks associated with a growing rat population will depend, in part, on improving ecologically based management strategies [50] that take into account how rats use urban spaces [5,6,51]. Despite large populations and a cosmopolitan distribution in cities across the globe, prominent knowledge gaps exist for developing ecologically based management strategies to control urban rat populations. These gaps can often be attributed to difficulty accessing urban rodent populations [5], which can be challenging to study owing to a complex mixture of social and logistical obstacles. Rat remains are commonly recovered during archaeological excavations and these remains are increasingly being preserved as part of the long-term curation of archaeological faunal assemblages, yet they remain underused. As this study demonstrates, the archaeological record can be used to study historical trends in the dynamics of rat dietary behaviour at a variety of scales and in spatio-temporal contexts that directly foreground many of the issues in rodent ecology being faced in today's modern cities.

## Supplementary Material

Tables S1 and S2 - S5; Figures S1 and S2

## Endnote

1 Unpublished *δ*^13^C values from nineteenth-century chickens (*Gallus gallus*) from sites in Upper Canada show that C_4_ plants, probably maize, were fed to some domestic fowl and suggest that at least some maize was kept on hand for this purpose.

## Data accessibility

All data presented in this study can be found in the electronic supplementary material.
