# Supplementary material for: Urban rats have less variable, higher protein diets
Source: Proc Biol Sci. 2018 Oct 17;285(1889):20181441. doi: 10.1098/rspb.2018.1441 (PMC6234891; doi:10.1098/rspb.2018.1441)
Supplement: Tables S1 and S2 - S5; Figures S1 and S2 [file rspb20181441supp1.pdf]

Table S1. Contextual information, ZooMS results, and isotopic, elemental, and collagen yeild values for samples analyzed in this study. For ZooMS resutls, 1 = confirmed *R. norvegicus* , 2 = inconclusive, 3 = not *Rattus*

IUBC No.	Site Name	Site No.	Site Type	Taxon	Element	Side	Cat. No.	Context summary	Col. Yld. (%)	ZooMS	$\delta^{13}C$ (‰)	$\delta^{15}N$ (‰)	C (%)	N (%)	C/N
2117	Trull	AlGq-67	Rural	*Rattus norvegicus*	Mandible	Right	2081	Feature 7, Layer C	17.9	*1*	-20.8	5.0	37.0	13.4	3.2
2118	Trull	AlGq-67	Rural	*Rattus norvegicus*	Mandible	Right	2081	Feature 7, Layer C	17.8	*1*	-20.5	5.6	36.8	13.4	3.2
2119	Trull	AlGq-67	Rural	*Rattus norvegicus*	Mandible	Right	2081	Feature 7, Layer C	5.1	*1*	-19.3	10.2	35.3	12.7	3.3
2125	Edgar	AlGu-196	Rural	*Rattus norvegicus*	Femur	Right	702	Feature 4, Q3	15.0	*2*	-21.1	5.0	39.9	14.4	3.2
2133	Henry	AhGw-123	Rural	*Marmota monax*	Humerus	Right	657	Feature 1	7.2	NA	-24.6	3.4	39.6	14.2	3.2
2134	Henry	AhGw-123	Rural	*Marmota monax*	Femur	Left	550	N498E197	15.2	NA	-18.0	4.8	40.4	14.6	3.2
2135	Henry	AhGw-123	Rural	*Procyon lotor*	Atlas	Axial	709	Feature 4	12.1	NA	-20.5	8.8	39.9	14.5	3.2
2143	Bishop's Block, House 3	AjGu-49	Urban	*Rattus norvegicus*	Innominate	Right	1858	Lot 29, House 3, Cistern 1	3.3	*1*	-19.2	10.0	38.3	13.3	3.3
2150	Bishop's Block, House 4	AjGu-49	Urban	*Rattus norvegicus*	Humerus	Left	3469	Lot 64, House 4. privy	10.7	2	-18.1	9.9	38.3	13.7	3.3
2151	Bishop's Block, House 4	AjGu-49	Urban	*Rattus norvegicus*	Humerus	Left	3469	Lot 64, House 4. privy	12.9	2	-19.4	10.3	39.7	14.1	3.3
2152	Bishop's Block, House 4	AjGu-49	Urban	*Rattus norvegicus*	Humerus	Left	3469	Lot 64, House 4. privy	12.0	2	-19.3	10.3	39.0	14.0	3.3
2153	Bishop's Block, House 4	AjGu-49	Urban	*Rattus norvegicus*	Mandible	Right	3469	Lot 64, House 4. privy	5.5	*1*	-17.9	9.6	37.2	13.4	3.2
2166	Bishop's Block, House 5	AjGu-49	Urban	*Rattus norvegicus*	Mandible	Left	NA	Lot 104, House 5, Privy	9.4	2	-18.8	10.5	39.8	14.3	3.2
2169	Bishop's Block, House 5	AjGu-49	Urban	*Rattus norvegicus*	Tibia	Right	4819	Lot 113, House 5, Stone privy	19.7	*1*	-20.5	8.8	40.3	14.4	3.3
2171	Bishop's Block, House 5	AjGu-49	Urban	*Rattus norvegicus*	Femur	Left	4656	Lot 114, House 5, Stone privy	13.6	*1*	-20.0	11.1	40.5	14.6	3.2
2173	Bishop's Block, House 5	AjGu-49	Urban	*Rattus norvegicus*	Tibia	Left	4878	Lot 117, House 5, Stone privy	13.1	*1*	-19.9	11.6	40.5	14.7	3.2
2179	Bishop's Block, House 5	AjGu-49	Urban	*Rattus norvegicus*	Tibia	Left	4878	Lot 117, House 5, Stone privy	17.7	2	-17.9	9.3	40.6	14.7	3.2
2180	Bishop's Block, House 5	AjGu-49	Urban	*Rattus norvegicus*	Tibia	Left	4878	Lot 117, House 5, Stone privy	18.1	*1*	-18.0	9.2	41.5	14.9	3.2
2181	Bishop's Block, House 5	AjGu-49	Urban	*Rattus norvegicus*	Tibia	Left	5152	Lot 123, House 5, Stone privy	10.0	2	-21.7	8.0	41.0	14.7	3.3
2191	Bishop's Block, House 6	AjGu-49	Urban	*Rattus norvegicus*	Femur	Right	5375	Lot 127, House 6, Cisten	11.0	*1*	-19.0	10.1	43.9	15.7	3.3
2200	Bishop's Block, House 6	AjGu-49	Urban	*Rattus norvegicus*	Innominate	Right	5447	Lot 130, House 6, privy	5.8	*1*	-19.2	10.2	40.0	14.4	3.2
2204	Bishop's Block, House 6	AjGu-49	Urban	*Rattus norvegicus*	Femur	Right	5521	Lot 131, House 6, privy	7.4	*1*	-19.1	9.9	38.5	14.0	3.2
2226	Ashbridge	AjGt-1	Rural	*Rattus norvegicus*	Femur	Left	1315	Contex 434, 5C8	8.6	*1*	-20.2	11.5	43.2	15.6	3.2
2227	Ashbridge	AjGt-1	Rural	*Rattus norvegicus*	Femur	Left	1062	Contex 368, 7D4	5.2	2	-17.6	9.1	43.0	15.5	3.2
2228	Ashbridge	AjGt-1	Rural	*Rattus norvegicus*	Mandible	Left	183	Contex 368, 7D4	18.0	*1*	-20.3	8.8	43.6	15.9	3.2
2231	Ashbridge	AjGt-1	Rural	*Rattus norvegicus*	Femur	Left	399	Contex 100, 8C3	9.1	*1*	-20.4	10.4	43.4	15.2	3.3
2232	Ashbridge	AjGt-1	Rural	*Rattus norvegicus*	Femur	Right	400	Contex 100, 8C3	4.8	*1*	-9.8	7.5	40.5	14.4	3.3
2234	Ashbridge	AjGt-1	Rural	*Rattus norvegicus*	Humerus	Left	244	Contex 37, 8D5	15.4	*1*	-19.3	8.4	39.9	14.3	3.2
2235	Ashbridge	AjGt-1	Rural	*Rattus norvegicus*	Femur	Left	222	Contex 34, 8D6	16.4	*1*	-17.7	7.1	40.5	14.6	3.2
2237	Ashbridge	AjGt-1	Rural	*Rattus norvegicus*	Mandible	Left	29	Contex 35, 8D7	13.6	*1*	-18.7	10.6	80.3	28.6	3.3
2238	Ashbridge	AjGt-1	Rural	*Rattus norvegicus*	Tibia	Left	231	Contex 35, 8D7	15.7	2	-20.7	5.6	40.0	14.4	3.2

IUBC No.	Site Name	Site No.	Site Type	Taxon	Element	Side	Cat. No.	Context summary	Col. Yld. (%)	ZooMS	$\delta^{13}C$ (‰)	$\delta^{15}N$ (‰)	C (%)	N (%)	C/N
2239	Ashbridge	AjGt-1	Rural	*Rattus norvegicus*	Tibia	Left	232	Contex 35, 8D7	12.1	*1*	-19.0	9.3	39.7	14.1	3.3
2240	Ashbridge	AjGt-1	Rural	*Rattus norvegicus*	Tibia	Left	233	Contex 35, 8D7	19.0	*1*	-20.4	8.9	41.0	14.6	3.3
2241	Ashbridge	AjGt-1	Rural	*Rattus norvegicus*	Femur	Right	110	Contex 21, 8F4	15.2	*1*	-14.8	9.6	41.2	15.0	3.2
2244	Ashbridge	AjGt-1	Rural	*Rattus norvegicus*	Scapula	Right	454	Contex 120, 10C7	18.5	*1*	-14.4	9.4	41.0	14.9	3.2
2245	Ashbridge	AjGt-1	Rural	*Procyon lotor*	Cranium	Axial	979	Contex 357, 10D7	8.2	NA	-18.6	8.4	39.3	14.6	3.2
2246	Ashbridge	AjGt-1	Rural	*Rattus norvegicus*	Humerus	Right	980	Contex 357, 10D7	16.5	*1*	-16.0	9.6	40.7	14.6	3.3
2247	Ashbridge	AjGt-1	Rural	*Rattus norvegicus*	Femur	Left	889	Contex 324, 10E4	13.0	*1*	-14.6	9.7	40.6	14.8	3.2
2248	Ashbridge	AjGt-1	Rural	*Rattus norvegicus*	Humerus	Right	2224	Contex 769, 11C3	12.1	*1*	-15.7	10.4	40.2	14.6	3.2
2252	Ashbridge	AjGt-1	Rural	*Rattus norvegicus*	Tibia	Right	2144	Contex 734, 11D4	17.7	*1*	-12.8	9.7	40.7	14.9	3.2
2254	Ashbridge	AjGt-1	Rural	*Rattus norvegicus*	Femur	Left	2698	Contex 896, 16A5	7.8	*1*	-19.8	9.5	40.0	14.3	3.3
2258	Ashbridge	AjGt-1	Rural	*Rattus norvegicus*	Humerus	Left	2773	Contex 919, 17B4	16.2	*1*	-20.5	10.0	41.3	14.8	3.3
2263	327-333 Queen	AjGu-63	Urban	*Marmota monax*	Cranium	Axial	16	Lot 38-01, Context 1	8.2	NA	-23.2	2.3	40.6	14.9	3.2
2267	Dolson	AkGx-80	Rural	*Marmota monax*	Mandible	Right	2099	Fill	8.1	NA	-22.3	4.5	39.5	14.5	3.2
2268	Dolson	AkGx-80	Rural	*Procyon lotor*	Tibia	Left	3449	300E 490N:18	16.7	NA	-20.1	8.4	40.4	14.9	3.2
2270	Dolson	AkGx-80	Rural	Rodentia	Femur	Left	2398	300E 505N:10	16.0	3	-18.7	7.8	40.5	15.0	3.2
2271	Dolson	AkGx-80	Rural	*Marmota monax*	Mandible	Left	1572	305E 490N:07	11.9	NA	-23.6	2.9	39.7	14.5	3.2
2273	Dolson	AkGx-80	Rural	*Marmota monax*	Mandible	Left	3597	305E 490N:12	16.3	NA	-23.6	2.8	40.0	14.6	3.2
2275	CH36	AlGr-315	Rural	*Rattus norvegicus*	Mandible	Left	265	Feature 1B	17.3	*1*	-20.5	8.6	41.4	15.3	3.2
2277	Hall	AlGw-68	Rural	*Marmota monax*	Mandible	Right	454	Context 123	17.8	NA	-20.3	6.6	41.5	15.1	3.2
2279	Hall	AlGw-68	Rural	*Rattus norvegicus*	Femur	Left	1088	Context 42	13.2	*1*	-19.9	8.6	40.5	14.7	3.2
2280	Hall	AlGw-68	Rural	*Rattus norvegicus*	Femur	Right	1351	Context 131	16.1	*1*	-20.1	6.9	41.4	15.1	3.2
2286	Graham	AjGs-370	Rural	*Rattus norvegicus*	Humerus	Right	NA		15.6	*1*	-21.1	7.5	41.9	13.8	3.5
2288	Graham	AjGs-370	Rural	*Procyon lotor*	Maxilla	Left	354	Context 222	10.1	NA	-21.9	6.0	42.0	14.0	3.5
2291	Lewis	AlGu-365	Rural	*Rattus norvegicus*	Femur	Right	1186	20W 9N (6)	19.2	*1*	-19.9	7.3	41.9	14.2	3.4
2292	Lewis	AlGu-365	Rural	*Rattus norvegicus*	Femur	Right	805	Lot 3	17.3	*1*	-20.6	7.9	42.4	14.4	3.4
2293	Lewis	AlGu-365	Rural	*Rattus norvegicus*	Femur	Right	805	Lot 3	16.4	*1*	-21.1	7.0	27.8	9.3	3.5
2294	Lewis	AlGu-365	Rural	*Rattus norvegicus*	Tibia	Left	878	28W 6N	13.1	*1*	-20.9	5.9	42.0	14.0	3.5
2297	Lewis	AlGu-365	Rural	*Marmota monax*	Humerus	Left	1139	40W 12N (3)	17.9	NA	-21.7	6.3	41.9	14.5	3.4
2298	Lewis	AlGu-365	Rural	*Procyon lotor*	Calcaneus	Left	824	56W 12N	10.6	NA	-21.1	7.0	41.1	13.9	3.4
2301	Lewis	AlGu-365	Rural	*Procyon lotor*	Astragalus	Right	1174	25W 0N	18.3	NA	-18.9	7.0	42.9	15.0	3.3
3433	Dollery	AjGu-81	Urban	*Rattus norvegicus*	Femur	Left	67	N500E195, Lot8	5.5	*1*	-19.8	10.0	41.2	14.3	3.4
3438	Dollery	AjGu-81	Urban	*Rattus norvegicus*	Femur	Left	135	N500E195, Lot8	9.2	*1*	-18.5	9.6	41.2	14.7	3.3

IUBC No.	Site Name	Site No.	Site Type	Taxon	Element	Side	Cat. No.	Context summary	Col. Yld. (%)	ZooMS	$\delta^{13}C$ (‰)	$\delta^{15}N$ (‰)	C (%)	N (%)	C/N
3448	Totonto General Hospital	AjGu-51	Urban	*Rattus norvegicus*	Tibia	Right	3	Lot 21	3.9	*1*	-19.4	11.8	41.4	14.8	3.3
3452	Totonto General Hospital	AjGu-51	Urban	*Rattus norvegicus*	Tibia	Right	372	N503E219, Lot 45	3.2	*1*	-19.9	11.4	42.1	14.9	3.3
3453	Totonto General Hospital	AjGu-51	Urban	*Rattus norvegicus*	Tibia	Right	404	N503E220, Lot 45	6.0	*1*	-19.4	10.5	40.5	13.8	3.4
3454	Totonto General Hospital	AjGu-51	Urban	*Rattus norvegicus*	Tibia	Right	427	N503E221, Lot 45	6.0	*1*	-19.3	10.4	42.3	14.0	3.5
3455	Totonto General Hospital	AjGu-51	Urban	*Rattus norvegicus*	Tibia	Right	940	Lot 52	6.6	*1*	-18.9	12.3	41.3	14.1	3.4
3456	Totonto General Hospital	AjGu-51	Urban	*Rattus norvegicus*	Tibia	Right	940	Lot 52	11.0	*1*	-19.1	11.4	41.6	14.2	3.4
3457	Totonto General Hospital	AjGu-51	Urban	*Rattus norvegicus*	Tibia	Right	2006	Lot 195	16.3	*1*	-20.4	10.9	42.0	14.8	3.3
3483	Totonto General Hospital	AjGu-51	Urban	*Rattus norvegicus*	Femur	Right	1502	N495E185, Lot 446	13.1	*1*	-21.0	9.2	42.1	15.0	3.3
3484	Totonto General Hospital	AjGu-51	Urban	*Rattus norvegicus*	Tibia	Left	307	Lot 289	15.3	*1*	-20.4	10.4	41.4	14.8	3.3
3485	Totonto General Hospital	AjGu-51	Urban	*Rattus norvegicus*	Tibia	Left	307	Lot 289	13.2	*1*	-18.8	9.5	42.3	15.1	3.3
3500	Trull	AlGq-67	Rural	*Rattus norvegicus*	Innominate	Left	564	Feature 7, Layer A	13.4	*1*	-20.8	5.6	41.8	14.5	3.4
3501	Trull	AlGq-67	Rural	*Rattus norvegicus*	Innominate	Left	564	Feature 7, Layer A	10.9	*1*	-20.7	11.5	42.2	14.0	3.5
3502	Trull	AlGq-67	Rural	*Rattus norvegicus*	Innominate	Left	564	Feature 7, Layer A	14.2	*1*	-20.9	5.4	42.3	14.7	3.4
3503	Trull	AlGq-67	Rural	*Rattus norvegicus*	Innominate	Left	564	Feature 7, Layer A	16.0	*1*	-21.1	6.2	42.2	14.4	3.4
3504	Trull	AlGq-67	Rural	*Rattus norvegicus*	Innominate	Left	564	Feature 7, Layer A	14.2	*1*	-21.0	11.1	42.2	14.3	3.4
3505	Trull	AlGq-67	Rural	*Rattus norvegicus*	Innominate	Left	564	Feature 7, Layer A	13.7	2	-20.9	10.6	42.0	14.4	3.4
3506	Trull	AlGq-67	Rural	*Rattus norvegicus*	Mandible	Right	2081	Feature 7, Layer C	5.9	*1*	-18.3	9.1	42.0	14.8	3.3
3507	Trull	AlGq-67	Rural	*Rattus norvegicus*	Mandible	Right	2081	Feature 7, Layer C	18.2	*1*	-20.4	10.4	42.0	13.5	3.6
3508	Trull	AlGq-67	Rural	*Rattus norvegicus*	Mandible	Right	2081	Feature 7, Layer C	11.7	*1*	-12.9	6.4	42.2	14.8	3.3
3512	Edgar	AlGu-196	Rural	*Rattus norvegicus*	Innominate	Right	662	Feature 4, Q1	14.4	*1*	-20.9	7.1	42.3	14.3	3.4
3513	Edgar	AlGu-196	Rural	*Rattus norvegicus*	Innominate	Right	542	Feature 4, Q1	13.1	*1*	-17.4	7.1	41.9	14.3	3.4
3514	Edgar	AlGu-196	Rural	*Rattus norvegicus*	Femur	Right	702	Feature 4, Q3	10.3	2	-21.5	5.4	41.7	14.2	3.4
3515	Edgar	AlGu-196	Rural	*Rattus norvegicus*	Femur	Right	702	Feature 4, Q3	10.7	*2*	-21.3	6.6	41.4	13.6	3.6
3553	Bishop's Block, House 3	AjGu-49	Urban	*Rattus norvegicus*	Mandible	Right	2781	Lot 59, House 3, Cistern 1	12.6	2	-20.4	9.7	42.0	14.3	3.4
3554	Bishop's Block, House 3	AjGu-49	Urban	*Rattus norvegicus*	Mandible	Left	2883	Lot 62, House 3, Cistern 2	12.5	*1*	-20.7	11.2	42.5	13.8	3.6
3555	Bishop's Block, House 3	AjGu-49	Urban	*Rattus norvegicus*	Mandible	Right	2883	Lot 62, House 3, Cistern 1	7.5	2	-21.5	10.8	41.4	13.5	3.6
3556	Bishop's Block, House 3	AjGu-49	Urban	*Rattus norvegicus*	Innominate	Right	1858	Lot 29, House 3, Cistern 1	12.2	*1*	-19.1	10.6	41.9	14.5	3.4
3557	Bishop's Block, House 3	AjGu-49	Urban	*Rattus norvegicus*	Innominate	Right	1858	Lot 29, House 3, Cistern 1	12.5	2	-19.6	9.9	41.2	14.2	3.4
3558	Bishop's Block, House 3	AjGu-49	Urban	*Rattus norvegicus*	Mandible	Left	2382	Lot 50, House 3, privy	10.3	*1*	-20.0	11.2	41.3	14.4	3.3
3559	Bishop's Block, House 3	AjGu-49	Urban	*Rattus norvegicus*	Mandible	Left	5873	Lot 50, House 3, privy	11.1	*1*	-19.8	11.8	41.2	13.8	3.5
3560	Bishop's Block, House 3	AjGu-49	Urban	*Rattus norvegicus*	Mandible	Left	2446	Lot 51, House 3, privy	13.4	*1*	-19.6	10.1	41.2	13.9	3.4
3561	Bishop's Block, House 3	AjGu-49	Urban	*Rattus norvegicus*	Mandible	Left	5858	Lot 50, House 3, privy	14.0	*1*	-19.4	10.2	42.0	14.2	3.4

IUBC No.	Site Name	Site No.	Site Type	Taxon	Element	Side	Cat. No.	Context summary	Col. Yld. (%)	ZooMS	$\delta^{13}C$ (‰)	$\delta^{15}N$ (‰)	C (%)	N (%)	C/N
3562	Bishop's Block, House 3	AjGu-49	Urban	*Rattus norvegicus*	Innominate	Right	1858	Lot 29, House 3, Cistern 1	10.9	*1*	-19.6	9.5	41.7	14.3	3.4
3563	Bishop's Block, House 4	AjGu-49	Urban	*Rattus norvegicus*	Innominate	Right	1944	Lot 35, House 4. privy	4.3	*1*	-20.3	9.3	40.0	13.3	3.5
3564	Bishop's Block, House 4	AjGu-49	Urban	*Rattus norvegicus*	Humerus	Left	3469	Lot 64, House 4. privy	12.8	*1*	-19.5	10.2	42.1	14.3	3.4
3565	Bishop's Block, House 5	AjGu-49	Urban	*Rattus norvegicus*	Femur	Right	4719	Lot 106, House 5, privy	3.8	*1*	-20.0	10.2	40.8	13.1	3.6
3566	Bishop's Block, House 4	AjGu-49	Urban	*Rattus norvegicus*	Humerus	Left	3668	Lot 64, House 4. privy	11.6	*1*	-19.8	10.3	42.0	14.0	3.5
3567	Bishop's Block, House 5	AjGu-49	Urban	*Rattus norvegicus*	Femur	Right	4719	Lot 106, House 5, privy	11.8	*1*	-19.9	10.7	41.8	13.8	3.5

Calibration and Analytical Uncertainty for Isotopic Measurements

Calibration, Accuracy, and Precision

Carbon and nitrogen isotopic and elemental compositions were determined using an Elementar Vario MICRO cube elemental analyzer coupled via continuous flow to an Isoprime isotope ratio mass spectrometer in the Archaeology Chemistry Laboratory at the University of British Columbia, Canada. Sample measurements were calibrated relative to VPDB ($\delta^{13}C$) and AIR ($\delta^{15}N$) [1]. Each analytical session included 8 or 9 aliquots each of USGS40 and USGS41 or USGS41a.

Table S2. Standard reference materials used for calibration of $\delta^{13}C$ relative to VPDB and $\delta^{15}N$ relative to AIR for the Delta V.

Standard	Material	Accepted $\delta^{13}C$ (‰, VPDB)	Accepted $\delta^{15}N$ (‰, AIR)
USGS40	Glutamic Acid	−26.389	−4.52
USGS41	Glutamic Acid	+37.626	+47.57
USGS41a	Glutamic Acid	+36.549	+47.23

The following internal standards were used to monitor accuracy and precision (Table S2). Each analytical session included 5 to 7 aliquots of at least two and more typically three check standards. The isotopic compositions reported here represent long term averages calibrated to VPDB and AIR with USGS40 and USGS41 or USGS41a: methionine (*n*=349), SRM-1 (*n*=461), SRM-2 (*n*=394), SRM-15 (*n*=27), and SRM-16 (*n*=27).

Table S3. Standard reference materials used to monitor internal accuracy and precision.

Standard	Material	Mean $\delta^{13}C$ (‰, VPDB)	Mean $\delta^{15}N$ (‰, AIR)
MET	Methionine	−28.60±0.08	−5.04±0.15
SRM-1	Caribou bone collagen	−19.36±0.11	+1.81±0.10
SRM-2	Walrus bone collagen	−14.76±0.12	+15.59±0.11
SRM-15	Deer bone collagen	−26.86±0.04	+6.90±0.07
SRM-16	Seal bone collagen	−14.80±0.06	+16.91±0.08

Table S4 summarizes the mean and standard deviation of carbon and nitrogen isotopic compositions for all check standards, as well as the standard deviation for all calibration standards – the mean of the calibration standard for an individual run is predetermined to calibrate the data. Note that means for calibration standards are not presented, as they are pre-determined to be equal to the known value.

RUN ID	Standard	n	$\delta^{13}C$			$\delta^{15}N$			%C			%N		
CN17-10	METH	7	-28.62	±	0.04	-5.10	±	0.11	39.60	±	0.94	9.36	±	0.20
CN17-10	SRM-1	6	-19.39	±	0.05	1.74	±	0.10	41.89	±	2.16	15.08	±	0.82
CN17-10	SRM-2	5	-14.79	±	0.05	15.49	±	0.04	41.45	±	2.32	15.02	±	0.83
CN17-10	USGS-40	9		±	0.03		±	0.05	40.82	±	0.00	9.52	±	0.00
CN17-10	USGS-41a	10		±	0.04		±	0.24	40.63	±	1.51	9.49	±	0.36
CN17-11	METH	7	-28.66	±	0.04	-5.07	±	0.11	39.97	±	0.84	9.31	±	0.27
CN17-11	SRM-1	6	-19.39	±	0.03	1.79	±	0.04	44.44	±	2.28	15.95	±	1.01
CN17-11	SRM-2	4	-14.81	±	0.03	15.54	±	0.04	44.81	±	2.43	16.31	±	0.90
CN17-11	USGS-40	9		±	0.04		±	0.09	40.82	±	0.00	9.52	±	0.00
CN17-11	USGS-41a	10		±	0.04		±	0.22	41.87	±	1.79	9.80	±	0.47
CN17-18	METH	7	-28.60	±	0.04	-5.02	±	0.13	40.86	±	1.76	9.36	±	0.29
CN17-18	SRM-1	6	-19.38	±	0.02	1.86	±	0.08	42.47	±	0.44	15.25	±	0.20
CN17-18	SRM-2	5	-14.80	±	0.03	15.62	±	0.04	42.64	±	0.26	15.38	±	0.13
CN17-18	USGS-40	9		±	0.02		±	0.18	40.82	±	0.00	9.52	±	0.00
CN17-18	USGS-41a	9		±	0.04		±	0.15	41.04	±	0.33	9.55	±	0.08
CN17-19	METH	7	-28.62	±	0.04	-4.94	±	0.03	39.78	±	0.68	9.40	±	0.22
CN17-19	SRM-1	6	-19.39	±	0.05	1.84	±	0.07	42.93	±	0.58	15.37	±	0.09
CN17-19	SRM-2	5	-14.79	±	0.02	15.56	±	0.06	42.92	±	0.91	15.43	±	0.20
CN17-19	USGS-40	9		±	0.03		±	0.14	40.82	±	0.00	9.52	±	0.00
CN17-19	USGS-41	9		±	0.09		±	0.07	41.93	±	0.45	9.74	±	0.04
CN17-20	METH	12	-28.62	±	0.08	-5.03	±	0.18	40.52	±	0.97	9.38	±	0.16
CN17-20	SRM-1	9	-19.39	±	0.13	1.77	±	0.12	42.71	±	0.45	15.30	±	0.16
CN17-20	SRM-2	5	-14.92	±	0.27	15.49	±	0.13	42.59	±	0.90	15.22	±	0.18
CN17-20	USGS-40	11		±	0.08		±	0.14	40.82	±	0.00	9.52	±	0.00
CN17-20	USGS-41	9		±	0.06		±	0.19	41.66	±	0.41	9.72	±	0.10

CN17-26	METH	7	-28.60	±	0.04	-5.03	±	0.04	40.09	±	1.05	9.41	±	0.27
CN17-26	SRM-1	6	-19.39	±	0.03	1.76	±	0.10	42.77	±	0.85	15.29	±	0.36
CN17-26	SRM-2	5	-14.84	±	0.06	15.56	±	0.02	42.20	±	0.41	15.21	±	0.21
CN17-26	USGS-40	9		±	0.04		±	0.05	40.82	±	0.00	9.52	±	0.00
CN17-26	USGS-41a	9		±	0.03		±	0.15	41.44	±	0.76	9.67	±	0.17
CN18-17	METH	7	-28.63	±	0.07	-5.04	±	0.07	40.82	±	1.80	9.47	±	0.21
CN18-17	SRM-15	7	-26.88	±	0.04	6.89	±	0.04	43.45	±	0.66	15.70	±	0.16
CN18-17	SRM-16	5	-14.79	±	0.03	16.88	±	0.05	43.89	±	0.44	15.88	±	0.16
CN18-17	USGS-40	11		±	0.05		±	0.05	40.82	±	0.00	9.52	±	0.00
CN18-17	USGS-41a	8		±	0.09		±	0.11	41.22	±	0.18	9.61	±	0.09

13% of samples were analyzed in duplicate. The average difference between duplicate pairs was 0.01 ‰ for δ^{13}C (range of −0.01 to 0.01) and 0.05 ‰ for δ^{15}N (range of −0.01 to 0.02).

Table S5 Results from Mann-Whitney-Wilcoxon's test comparisons of δ^{15}N values from 10 sets of 20 randomly selected samples from rural and urban rat groups.

Test No.	W=	*p*=
1	50	<0.000
2	148	0.163
3	57	<0.000
4	53	<0.000
5	93	0.004
6	33	<0.000
7	89	0.003
8	97	0.006
9	25	0.006
10	82	0.002

ZooMS

Figure S1 - MALDI-ToF mass spectra of collagen peptide mass fingerprints separated into 10% and 50% acetonitrile (in 0.1% TFA) fractions, with the primary diagnostic marker for brown rat marked by the arrow (zoomed in on inset).

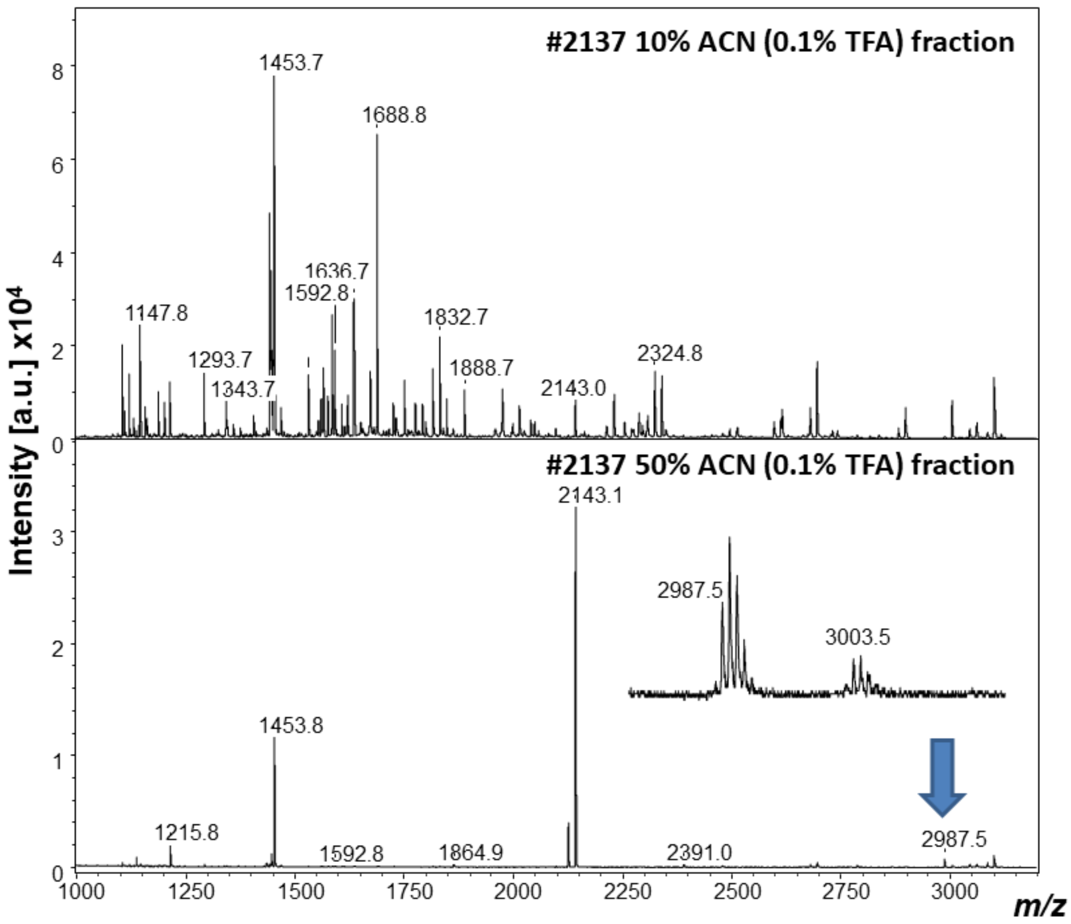

Figure S2. Stable carbon and nitrogen isotope values for historical archaeological rats and other animals analyzed in this study. Livestock data are from [2] and include both cattle and pig samples from a range of coeval historical archaeological sites (and in some case the same sites as rats) in the local region. As per [2], although technically omnivorous, pigs have a low mean $\delta^{15}N$ value relative to cattle and are therefore considered as part of the local herbivorous livestock baseline.

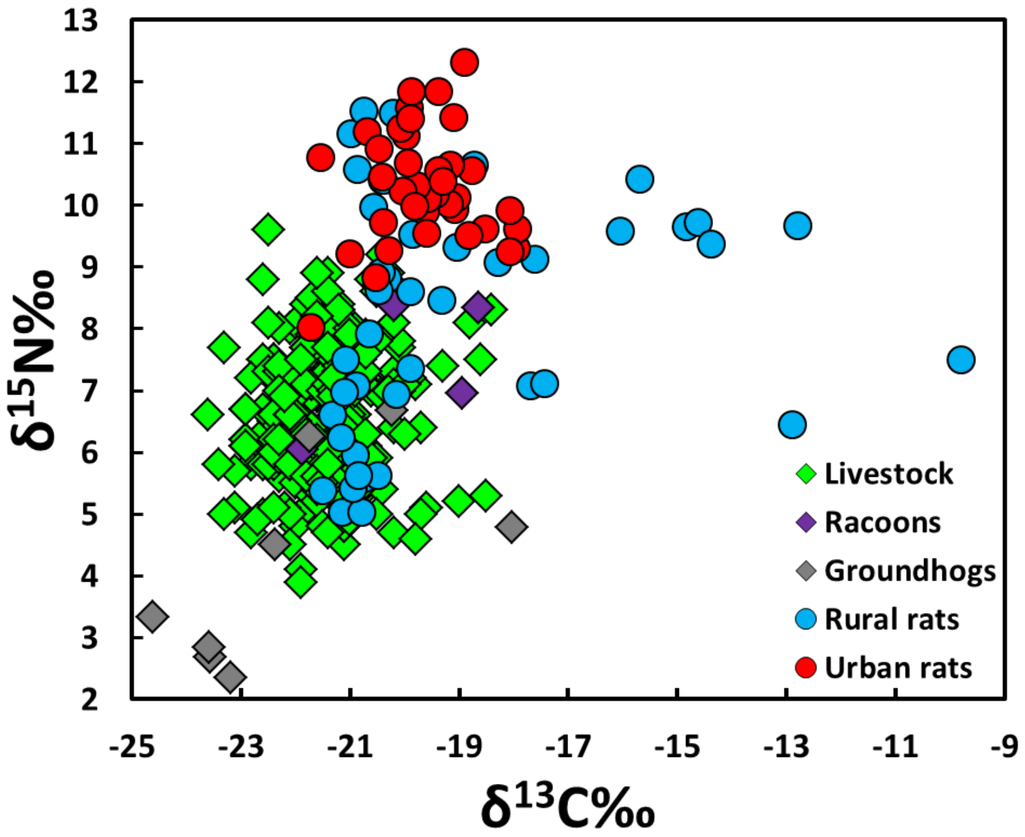

References

[1] Qi, H., Coplen, T. B., Geilmann, H., Brand, W. A. & Böhlke, J. 2003 Two new organic reference materials for δ13C and δ15N measurements and a new value for the δ13C of NBS 22 oil. *Rapid Communications in Mass Spectrometry* **17**, 2483-2487.

[2] Guiry, E., Szpak, P. & Richards, M. P. 2017 Isotopic analyses reveal geographical and socioeconomic patterns in historical animal trade between predominantly wheat- and maize-growing agricultural regions in eastern North America. *American Antiquity* **82**, 341-352.
